# Nonfatal self-injury in the first year after surgery: a population-based epidemiologic study

**DOI:** 10.1016/j.bja.2025.12.027

**Published:** 2026-01-22

**Authors:** Calvin Diep, Julian F. Daza, Sandra Lee, Christopher Witiw, Simone Vigod, Duminda N. Wijeysundera, Karim S. Ladha

**Affiliations:** 1 Department of Anesthesiology and Pain Medicine, University of Toronto, Toronto, ON, Canada; 2 Institute of Health Policy, Management and Evaluation, Dalla Lana School of Public Health, University of Toronto, Toronto, ON, Canada; 3 Division of General Surgery, Department of Surgery, University of Toronto, Toronto, ON, Canada; 4 Temerty Faculty of Medicine, University of Toronto, Toronto, ON, Canada; 5 Division of Neurosurgery, Department of Surgery, University of Toronto, Toronto, ON, Canada; 6 Division of Neurosurgery, Department of Surgery, St. Michael’s Hospital, Toronto, ON, Canada; 7 Department of Psychiatry, University of Toronto, Toronto, ON, Canada; 8 Department of Psychiatry, Women’s College Hospital, Toronto, ON, Canada; 9 Department of Anesthesia, St. Michael’s Hospital, Toronto, ON, Canada; 10 Department of Anesthesia and Pain Management, Toronto Western Hospital, Toronto, ON, Canada

**Keywords:** nonfatal self-injury, perioperative outcomes, postoperative outcomes, psychological distress, surgery

## Abstract

**Background:**

Recovery after surgery can be psychologically distressing, putting patients at risk of suicidal behaviours such as nonfatal self-injury (NFSI). We evaluated the incidence and identified factors associated with NFSI after surgery.

**Methods:**

Linked administrative databases from New York state were used to identify adults (≥18 yr) undergoing a broad range of surgical procedures between 2016 and 2018. The primary outcome was presentation to an emergency department or hospitalisation for NFSI within 365 days of discharge from the index surgical admission. The incidence was compared with two comparator groups using doubly robust estimation: percutaneous coronary interventions and cataract procedures. Patient- and surgery-related factors associated with NFSI after surgery were determined using Cox models.

**Results:**

Among 1 165 881 patients, 824 presented with NFSI within the year after surgery (incidence 7.07 per 10 000 patients). This was higher than the incidence in the percutaneous coronary interventions group (4.88 per 10 000; adjusted hazard ratio, 1.55; 95% confidence interval, 1.06–2.27) and the cataract group (1.52 per 10 000; adjusted hazard ratio, 1.48; 95% confidence interval, 1.04–2.09). Risk factors for NFSI were younger age, White race, lower income, rural residence, previous psychiatric history, and nonelective surgery.

**Conclusions:**

Select patient groups were more susceptible to NFSI after surgery. The true burden of suicidal behaviours and severe psychological distress after surgery is likely underestimated by analyses of administrative data. Future studies should investigate psychological distress after surgery using validated instruments.

**Editor’s key points**

• The incidence and specific factors associated with nonfatal self-injury after surgery have not been systematically assessed.
• In this population-based study of administrative data from New York state, the authors estimated the incidence and risk factors associated with nonfatal self-injury in the first year after a broad range of surgical procedures.
• Young adults and those with a history psychiatric disease were particularly susceptible with an overall incidence of about seven in 10 000, although the true incidence is likely greater.

Surgery can be psychologically challenging, including preparations, undergoing the procedure, and recovery.^1^ Although most research on surgical and perioperative outcomes has focused on mitigating the physiologic effects of surgery, a complete recovery also entails alleviation of various ‘mental strains’ including worries related to the need for repeat procedures, development of chronic pain, and poor functional recovery.^1^^,^^2^ Such strains can lead to significant psychological distress, with studies showing that up to 30% of adult patients report persistent moderate to severe psychological distress in the weeks and months after surgery.^3^^,^^4^

Persistent psychological distress is a well-established antecedent of suicidal behaviour (e.g. suicidal ideation, self-harm, or suicide attempt).^5^ Previous studies have shown that stressful and potentially life-altering health-related events such as critical illness or cancer diagnoses are associated with increased risk of behaviours such as nonfatal self-injury (NFSI).^6^^,^^7^ Similarly, undergoing a major surgical procedure can plausibly be associated with increased psychological distress and progression to NFSI. However, this has not yet been reported. We conducted this exploratory, hypothesis-generating study guided by two objectives: (i) to evaluate the incidence of NFSI after surgery in a population-based cohort undergoing a broad range of surgical procedures, and (ii) to identify baseline patient- and surgery-related factors associated with NFSI after surgery.

## Methods

This study was conducted and reported in accordance with the REporting of studies Conducted using Observational Routinely-collected health Data (RECORD) guidelines.^8^ The study objectives, cohort definitions, and analytic approach were determined *a priori*.

### Data sources

We accessed linked discharge records from the State Inpatient Database (SID), State Ambulatory Surgery Database (SASD), and State Emergency Department Database (SEDD) from New York state for the years 2016–2019. These data are available through the Healthcare Cost and Utilization Project (HCUP). As these data are fully de-identified and publicly available, this study was exempt from institutional review board (IRB) review and approval.

New York state was selected given the validity of the linkage variable between the databases, which allows longitudinal follow-up for each patient across multiple visit types and centres. State-level data from HCUP are representative of the state’s census and capture all visits to inpatient hospitals, ambulatory surgical centres, and emergency departments except specialised centres that provide only psychiatric services.

### Study population

The analytic cohort was comprised of adult patients (≥18 yr old) who underwent an eligible surgical procedure from 2016 to 2018. All procedures performed at an inpatient centre are coded in the SID using codes from the Procedure Coding System of the International Classification of Diseases, Tenth Revision (ICD-10-PCS), and procedures performed at an ambulatory surgical centre are coded in the SASD using Current Procedural Terminology (CPT) codes. Eligible procedure codes were selected to include a broad range of procedures across surgical subspecialties performed in an operating room requiring at least regional or general anaesthesia (i.e. not sedation alone). These included breast, ear-nose-throat (otolaryngologic), general surgery, gynaecologic, neurosurgery (intracranial), orthopaedic, spine, thoracic, urologic, and vascular procedures. A detailed list and categorisation of included codes are provided in Supplementary Appendix A. Obstetrics-related procedures (e.g. Caesarean delivery, cervical cerclage, dilatation and curettage, instrument-assisted deliveries), cardiac procedures, and solid organ transplants were excluded as these populations are known to have distinct epidemiology of adverse mental health outcomes related to the specific physiological changes, indications for surgery, or surgical outcomes.^9^, ^10^, ^11^

### Outcome measures and timeline

The primary outcome was NFSI in the first year (365 days) after hospital discharge after the index eligible surgical procedure. NFSI was defined as an emergency department presentation or hospital admission for reasons such as intentional poisonings, intentional physical self-harm, or nonfatal suicide attempts by other means. Suicidal ideation was intentionally excluded from the outcome definition as only patients with the most severe forms would be captured presenting to care, potentially introducing selection bias. The complete list of eligible ICD-10 Clinical Modification (CM) codes is provided in Supplementary Appendix B. These codes have previously been validated for identifying causes and mechanisms of self-harm in other administrative database studies, although they are known to underestimate the true incidence as many individuals who self-harm do not seek medical care.^12^, ^13^, ^14^ For patients who presented with NFSI more than once in the 1-yr period after surgery, only the first instance was considered in analyses.

### Predictor variables

Patient characteristics included age (categorised as younger [18–39 yr], middle-aged [40–64 yr], and older [≥65 yr]), biological sex (male or female), and race (White, Black, Hispanic, other). Socioeconomic characteristics included median household income by ZIP code (categorised in quartiles), health insurance coverage (private, Medicare, Medicaid, none/other), and location of residence (rural or urban). Medical comorbidity burden was measured using the Charlson Comorbidity Index (CCI) from ICD-10-CM codes. CCI was categorised as 0, 1–2, or ≥3.^15^ Pre-existing anxiety, mood/affective, psychotic, or substance use disorders, and previous suicidality (defined as a history of hospital presentation for suicidal ideation or NFSI) were identified using ICD-10-CM codes and included as baseline psychiatric characteristics (Supplementary Appendix C). All patient-related predictor variables were measured at the index surgical visit. In addition, surgery characteristics such as procedure urgency (elective *vs* nonelective) and subspecialty (breast, otolaryngologic, general surgery, gynaecology, neurosurgery, orthopaedic, spine, thoracic, urology, and vascular) were included. Amongst the subspecialties, spine was assigned the reference level as this population has been previously shown to have a high burden of psychological distress after surgery.^16^^,^^17^ Post-surgical variables (e.g. length of stay, complications, additional surgeries) were not considered in this study owing to the risk of introducing post-exposure bias.

### Statistical analysis

Descriptive statistics were used to summarise baseline characteristics and outcome measures. Standardised mean differences (SMDs) were used to compare patients with and without NFSI.

To address the first study objective, the incidence of NFSI was estimated in the primary surgical cohort. Then, the incidence was compared with two appropriate comparator groups: percutaneous intervention (PCI) and cataract surgery. The procedural codes used are outlined in Supplementary Appendices D and E, respectively. Two matched analytic datasets were then constructed, excluding patients who underwent both an eligible surgery and the comparator procedure within the follow-up period to reduce cross-contamination. Adjusted hazards of the outcome between the surgical and comparator cohorts were estimated using a doubly robust approach. This involved matching patients in the two cohorts using 1:1 nearest neighbour propensity score matching (incorporating all aforementioned baseline covariates, except surgical subspecialty) without replacement and a caliper of 0.05, then including any covariates with residual imbalance (SMD >10%) into a multivariable Cox model. Estimates produced from this approach are robust to model misspecification in either the propensity score model or the survival model.^18^

To address the second study objective, Cox proportional hazards models were fitted to assess the association between each of the included covariates and the hazard of NFSI. Each predictor variable was first evaluated for its crude (unadjusted) association with the outcome. A Kaplan–Meier curve of NFSI-free time after surgery was plotted for the population stratified by each predictor variable. All predictor variables were then included as covariates in a multivariable Cox model to evaluate adjusted associations with the outcome. In a sensitivity analysis to assess the potential impact of the competing risk of death on the primary outcome of NFSI, any deaths in the linked databases during the same observation period were captured. To determine the crude and adjusted associations between each predictor and NFSI while accounting for the occurrence of competing outcomes, Fine and Gray models were fitted.^19^ Point estimates from these models represented the subdistribution hazard ratio (sdHR), in contrast to the cause-specific hazard ratio (csHR) from Cox models. Whereas cause-specific hazards are useful for studying the aetiology of an outcome of interest, subdistribution hazards are appropriate for predicting the risk of an outcome of interest while accounting for the occurrence of truncating events (e.g. death).

No predictor variables had >1% missing values in the overall sample; thus, complete case analyses were conducted. Proportional hazards assumptions in all Cox models were assessed using Schoenfeld residuals. Variance inflation factors were examined to assess collinearity, with no concerning values observed. All statistical tests were two sided with significance level defined at *P*<0.05. Analyses were performed using the *survival* package in R v3.5.2 (R Core Team, Vienna, Austria). Sample size was based on the available data and no *a priori* power calculations were performed.^20^ Given the exploratory nature of this analysis, no adjustments were made for multiple comparisons.

## Results

A total of 1 165 881 unique patients underwent at least one eligible surgical procedure in New York state between 2016 and 2018. Baseline characteristics are presented in Table 1. In this cohort, 824 patients presented for NFSI at least once in the first 365 days after discharge from an index surgery (564 presented to emergency departments and 260 were admitted to hospital). The median (interquartile range [IQR]) time to NFSI was 168 (77–256) days. Most index NFSI events were related to poisoning by medications (60.9%), and the second most common specified reason was injury by a sharp object (13.0%); a full breakdown of reasons is presented in Supplementary Appendix F. Multiple presentations for NFSI were observed for 88 patients (Supplementary Appendix G), with one patient having a maximum of seven presentations in the year after surgery.

**Table 1:** Baseline cohort characteristics, stratified by the occurrence of NFSI in the first year after surgery. All values are presented as *n* (%). ENT, ear, nose, and throat (otolaryngology); NFSI, nonfatal self-injury; SMD, standardised mean difference.

	Overall sample	NFSI within 1 yr		Absolute SMD
	(*N*=1 165 881)	Yes **(*n*=824)**	No **(*n*=1165057)**	
Age (yr)				0.678
18–39	392655 (33.7)	366 (44.4)	232686 (20.0)	
40–64	540174 (46.3)	366 (44.4)	539808 (46.3)	
65+	233052 (20.0)	92 (11.2)	392563 (33.7)	
Female sex	727106 (62.4)	569 (69.1)	726537 (62.4)	0.141
Race				0.190
White	736848 (63.2)	568 (68.9)	736280 (63.2)	
Black	128721 (11.0)	90 (10.9)	128631 (11.0)	
Hispanic	126806 (10.9)	93 (11.3)	126713 (10.9)	
Other	173506 (14.9)	73 (8.9)	173433 (14.9)	
Income (quartile)				0.328
1 (lowest)	255419 (22.1)	251 (30.7)	255168 (22.1)	
2	285204 (24.7)	240 (29.4)	284964 (24.6)	
3	304436 (26.3)	203 (24.8)	304233 (26.3)	
4 (highest)	311809 (27.0)	123 (15.1)	311686 (27.0)	
Missing	9013	7	9006	
Health insurance				0.604
Private	515949 (44.3)	241 (29.2)	515708 (44.3)	
Medicare	382995 (32.9)	177 (21.5)	382818 (32.9)	
Medicaid	199049 (17.1)	355 (43.1)	198694 (17.1)	
Uninsured/other	67192 (5.8)	51 (6.2)	67141 (5.8)	
Missing	696	0	696	
Rural location	98182 (8.4)	129 (15.7)	98053 (8.4)	0.224
Missing	1130	1	1129	
Charlson Comorbidity Index				0.096
None (0 points)	655862 (56.3)	455 (55.2)	655407 (56.3)	
Mild (1–2)	424076 (36.4)	325 (39.4)	423751 (36.4)	
Moderate/severe (3+)	85943 (7.4)	44 (5.3)	85899 (7.4)	
Anxiety disorder	114307 (9.80)	219 (26.6)	114088 (9.8)	0.446
Mood disorder	97440 (8.4)	237 (28.8)	97203 (8.3)	0.544
Psychotic disorder	5101 (0.4)	26 (3.2)	5075 (0.4)	0.206
Substance use disorder	123765 (10.6)	250 (30.3)	123515 (10.6)	0.505
History of suicidality	880 (0.1)	16 (1.9)	864 (0.1)	0.188
Nonelective surgery	243557 (20.9)	230 (27.9)	243327 (20.9)	0.164
Missing	571	0	571	
Type of surgery				0.247
Breast	103208 (8.8)	95 (11.5)	109635 (9.4)	
ENT	31744 (2.7)	51 (6.2)	103157 (8.8)	
General surgery	388705 (33.3)	16 (1.9)	31728 (2.7)	
Gynaecology	144047 (12.4)	323 (39.2)	388382 (33.3)	
Neurosurgery	15504 (1.3)	130 (15.8)	143917 (12.4)	
Orthopaedic	275037 (23.6)	14 (1.7)	15490 (1.3)	
Spine	109730 (9.4)	143 (17.4)	274894 (23.6)	
Thoracic	29276 (2.5)	16 (1.9)	29260 (2.5)	
Urology	43393 (3.7)	24 (2.9)	43369 (3.7)	
Vascular	25237 (2.2)	12 (1.5)	25225 (2.2)	

In this primary surgical cohort, the risk of NFSI was 7.07 per 10 000 patients (95% confidence interval [95% CI], 6.58–7.55) in the first year after surgery. In the same period, 91 989 adults underwent PCI, of whom 7907 were excluded for also undergoing an eligible surgical procedure during the study period. NFSI in the year after PCI occurred in 41 patients, an incidence rate of 4.88 per 10 000 (95% CI, 3.09–5.82). Compared with the PCI cohort, those in the primary cohort had a crude HR of the outcome of 1.46 (95% CI, 1.06–1.99). After doubly robust estimation (see Supplementary Appendices H and I for propensity score-matched cohorts and multivariable Cox model, respectively), the adjusted HR (aHR) was 1.55 (95% CI, 1.06–2.27). Similar estimates were found when comparing the primary cohort with a cohort of 395 011 adults undergoing cataract surgery in the same period. Of these, 27 709 were excluded for also undergoing an eligible surgical procedure during the study period. NFSI in the year after cataract surgery was experienced by 56 patients, an incidence rate of 1.52 per 10 000 (95% CI, 1.13–1.92). Compared with the cataract cohort, those in the primary cohort had a crude HR of the outcome of 4.71 (95% CI, 3.59–6.17). After doubly robust estimation (see Supplementary Appendices J and K for propensity score-matched cohorts and multivariable Cox model, respectively), the aHR was 1.48 (95% CI, 1.04–2.09).

The incidence of the outcome for each subgroup of each predictor variable is presented in Table 2. Crude associations from bivariate models of each predictor variable with the outcome are also presented in Table 2. The proportions of each subgroup without NFSI after surgery are plotted as Kaplan–Meier curves in Supplementary Appendices L–Y.

**Table 2:** Associations between predictor variables and nonfatal self-injury in the first year after surgery from Cox survival models. Bolded estimates are statistically significant at *P*<0.05. ENT, ear, nose, and throat (otolaryngology); HR, hazard ratio; 95% CI, 95% confidence interval.

	Rate per 10 000	Crude HR (95% CI)	Adjusted HR (95% CI)
Overall sample	7.07		
Age (yr)			
18–39	15.70	**6.71 (5.34–8.43)**	**8.93 (6.52–12.21)**
40–64	6.78	**2.89 (2.30–3.64)**	**3.81 (2.86–5.06)**
65+	2.34	1 (ref)	1 (ref)
Sex			
Female	7.83	**1.35 (1.16–1.56)**	1.16 (0.98–1.37)
Male	5.81	1 (ref)	1 (ref)
Race			
White	7.71	**1.83 (1.44–2.34)**	**2.01 (1.56–2.59)**
Black	6.99	**1.66 (1.22–2.26)**	1.34 (0.98–1.84)
Hispanic	7.33	**1.74 (1.28–2.37)**	1.25 (0.92–1.71)
Other	4.21	1 (ref)	1 (ref)
Income (quartile)			
1 (lowest)	9.83	**2.49 (2.01–3.09)**	**1.39 (1.10–1.76)**
2	8.42	**2.13 (1.72–2.65)**	**1.34 (1.07–1.68)**
3	6.67	**1.69 (1.35–2.11)**	**1.35 (1.07–1.69)**
4 (highest)	3.94	1 (ref)	1 (ref)
Health insurance			
Private	4.67	1 (ref)	1 (ref)
Medicare	4.62	0.99 (0.82–1.20)	**2.35 (1.84–3.01)**
Medicaid	17.84	**3.82 (3.24–4.50)**	**2.83 (2.37–3.38)**
Uninsured/other	7.59	**1.63 (1.20–2.20)**	**1.52 (1.12–2.08)**
Patient location			
Urban	6.51	1 (ref)	1 (ref)
Rural	13.14	**2.02 (1.67–2.44)**	**1.40 (1.14–1.72)**
Charlson Comorbidity Index			
None (0 points)	6.94	1 (ref)	1 (ref)
Mild (1–2)	7.66	1.10 (0.96–1.27)	**1.19 (1.02–1.39)**
Moderate/severe (3+)	5.12	0.74 (0.54–1.01)	0.92 (0.65–1.29)
Anxiety disorder			
No	5.75	1 (ref)	1 (ref)
Yes	19.16	**3.33 (2.86–3.89)**	**1.74 (1.45–2.08)**
Mood disorder			
No	5.49	1 (ref)	1 (ref)
Yes	24.32	**4.43 (3.81–5.15)**	**2.48 (2.07–2.97)**
Psychotic disorder			
No	6.88	1 (ref)	1 (ref)
Yes	50.97	**7.43 (5.03–10.98)**	**2.97 (1.97–4.48)**
Substance use disorder			
No	5.51	1 (ref)	1 (ref)
Yes	20.20	**3.67 (3.16–4.26)**	**1.94 (1.65–2.29)**
History of suicidality			
No	6.94	1 (ref)	1 (ref)
Yes	181.82	**26.47 (16.14–43.41)**	**5.22 (3.09–8.84)**
Urgency of surgery			
Elective	6.44	1 (ref)	1 (ref)
Nonelective	9.44	**1.47 (1.26–1.71)**	**1.21 (1.02–1.42)**
Type of surgery			
Breast	4.94	**0.57 (0.41–0.80)**	0.80 (0.56–1.14)
ENT	5.04	**0.58 (0.34–0.99)**	0.77 (0.45–1.31)
General surgery	8.31	0.96 (0.76–1.21)	0.93 (0.74–1.18)
Gynaecology	9.02	1.04 (0.80–1.36)	0.86 (0.65–1.14)
Neurosurgery	9.03	1.04 (0.60–1.83)	0.95 (0.53–1.71)
Orthopaedic	5.20	**0.60 (0.46–0.78)**	0.90 (0.69–1.17)
Spine	8.66	1 (ref)	1 (ref)
Thoracic	5.46	0.63 (0.37–1.07)	0.57 (0.31–1.05)
Urology	5.53	**0.64 (0.41–0.99)**	0.95 (0.60–1.51)
Vascular	4.76	0.55 (0.30–1.00)	0.81 (0.44–1.51)

In the multivariable Cox model with all predictors included (Table 2), covariates associated with the outcome included being younger (aHR, 8.93; 95% CI, 6.52–12.21) or middle-aged (aHR, 3.81; 95% CI, 2.86–5.06) compared with older adults; White race (aHR, 2.01; 95% CI, 1.56–2.59) compared with ‘other’; being in any income quartile other than the highest quartile (range of aHRs, 1.35–1.39 for different quartiles); not having private health insurance (range of aHRs, 1.52–2.83, depending on type of insurance); rural location (aHR, 1.40; 95% CI, 1.14–1.72); CCI score of 1–2 (aHR, 1.19; 95% CI, 1.01–1.39) compared with a score of 0; any psychiatric comorbidities such as pre-existing anxiety (aHR, 1.74; 95% CI, 1.45–2.08), mood disorders (aHR, 2.48; 95% CI, 2.07–2.97); psychotic disorders (aHR, 2.97; 95% CI, 1.97–4.48), substance use disorders (aHR, 1.94; 95% CI, 1.65–2.29), or previous suicidality (aHR, 5.22; 95% CI, 3.09–8.84); and nonelective surgery (aHR, 1.21; 95% CI, 1.02–1.42).

During the same observation period, there were 21 163 (1.8%) deaths in the included sample, with a median (IQR) time to death of 36 (0–152) days after index surgical discharge. For each predictor variable, the cumulative incidence of NFSI and the crude and adjusted associations with the outcome, while accounting for the competing risk of death, are presented in Supplementary Appendix Z. Overall, the parameter estimates from the bivariate and multivariable Fine and Gray models were similar to those from the corresponding Cox models, suggesting that the number of people who experienced the competing risk of non-suicide death did not have a substantial impact on the incidence of NFSI for the set of people at risk.

## Discussion

Using population-based data from New York state, we found the incidence of NFSI in the first year after surgery to be about seven per 10 000 patient-years. Patients at greater risk for NFSI after surgery included those with younger age, female sex, White race, lower income, rural location, previous psychiatric history, or nonelective surgery. These findings are hypothesis-generating and can be used to inform future investigations of risk factors and prediction models for severe psychological distress after surgery.

No previous studies have described the incidence of NFSI after surgery. The incidence of NFSI after surgery was instead compared with matched patients in the same dataset undergoing less invasive procedures such as PCI and cataract surgery, in which the incidence was expectedly lower. In addition, the incidence of NFSI in this study can be contextualised with similar outcomes after other major health-related events such as cancer diagnoses and hospitalisations reported in other studies. For example, one study of cancer surgery patients in the USA reported a cumulative incidence of completed suicide of 0.08% over a median 4.6 yr, with 21% of cases occurring in the first year after surgery.^21^ However, they did not report outcomes other than completed suicide, which is a much more tragic but rare psychiatric outcome compared with NFSI, and represents the most severe psychiatric distress. One population-based study from Ontario, Canada, reported a cumulative incidence of NFSI in the first 5 yr after a cancer diagnosis (but not necessarily after cancer-related surgery) of 0.27%.^6^ Another study of intensive care unit survivors from Ontario, Canada, reported a cumulative incidence of self-harm of 32.8 per 10 000 patient-years,^7^ greater than the 7.1 per 10 000 patient-years observed in our cohort. Our findings are therefore plausible in the context of existing literature; undergoing and recovering from the average surgery can be thought of as less mentally distressing compared with cancer diagnosis and treatment and intensive care admissions, especially considering that most surgeries are elective in nature and perioperative complications are not anticipated.

The patient-related factors associated with NFSI in this study are similar to risk factors for suicidality in the nonsurgical, broader population. Patients with any psychiatric history in our study had the highest incidence of NFSI, which is consistent with existing knowledge.^22^ Younger age, White race, lower income, and rural location are also established risk factors for suicidality.^23^^,^^24^ Nonelective surgery, usually after traumatic accidents, has also been described as exacerbating psychological stress.^25^^,^^26^ Although patients undergoing spine procedures are known to have high levels of perioperative anxiety and depression given the degenerative nature of their diseases and challenges of rehabilitation after surgery,^16^^,^^17^ we did not find a consistent signal that spine surgery was more (or less) associated with NFSI than other types of surgery.^27^ The increased hazard of spine surgery compared with other types observed in crude models was explained by other confounding patient factors (e.g. patient characteristics or baseline psychiatric history) in multivariable modelling.

The true impact of preparing for, undergoing, and recovering from surgery on psychological distress remains unknown. In this study, we could only capture NFSI from patients who presented to emergency departments or were hospitalised, which is certainly an underestimation of the true incidence as not all patients who self-harm present to hospital. Furthermore, suicidal behaviours (e.g. ideation, self-harm, and suicide attempt) represent only the extreme end of the spectrum of psychological distress. Because patients experiencing severe psychological distress are known to avoid presenting to care,^28^ the true burden of psychological distress after surgery is expected to be greater than the measured incidence of NFSI from administrative discharge databases such as in this study. Future prospective studies using validated and frequent measures of anxiety and depression are needed to better understand patient experiences and factors contributing to psychological distress and suicidal behaviours after surgery, especially because these are strong predictors of future suicidal behaviours and attempts.^29^ More specifically, the mediating effects of postoperative factors such as length of stay, complications, acute pain, and disability on postoperative development of psychological distress warrant further investigation.

There are several key study limitations to consider. Firstly, detecting either suicidal behaviours or deaths using hospital discharge data is restricted to diagnostic codes and relies on presentations to hospital. This approach is known to have low sensitivity and predictive value and underestimates the true incidence of these events as an unknown proportion of affected patients never present to care.^30^ Secondly, our data were limited to a single state in the USA. Patients who underwent surgery in New York but presented for NFSI in another state, or *vice versa*, would be missed. However, most patients can reasonably be expected to both undergo surgery and present for acute care in the jurisdiction where they reside. Similarly, surgical history before the study period could not be comprehensively captured across all care settings and therefore was not included to avoid misclassification. Thirdly, administrative databases cannot capture all confounder or mediator variables associated with mental health outcomes such as baseline symptom burden, engagement in psychiatric interventions (e.g. medications, psychotherapy), resilience, and social factors (e.g. marital status, social support).

### Conclusions

In this population-based study, we estimated the incidence and risk factors associated with NFSI in the first year after a broad range of surgical procedures. Certain groups such as young adults and those with psychiatric history were particularly susceptible. Undergoing and recovering from surgery can be psychologically distressing, putting patients at risk of severe and potentially tragic outcomes such as suicidal behaviours. Future prospective investigations are needed to explore predictors and trajectories of severe psychological distress after surgery, as the true incidence is likely greater than what can be detected using administrative data alone.

## Authors’ contributions

Conceptualisation, methodology, writing—review and editing: all authors

Software, validation, formal analysis, investigation, data curation, writing—original draft, visualisation, project administration: CD

Resources: CD, KSL

Supervision: KSL

## Funding

University of Toronto (Canada Graduate Scholarship to CD); Department of Anesthesiology and Pain Medicine at the University of Toronto (Merit Award to DNW and KSL); Endowed Chair in Translational Anesthesiology Research at St. Michael’s Hospital and the University of Toronto (DNW); Canadian Anesthesiologists’ Society (Career Scientist Award to KSL); the Evelyn Bateman Recipe Chair in Ambulatory Anesthesia and Women’s Health at Women’s College Hospital (KSL).

## Declarations of interest

SV has previously received royalties from UpToDate Inc. for authorship of materials related to depression and pregnancy, unrelated to the submitted work. KSL has previously received advisory board fees from Vectura Fertin Pharma and Merck, unrelated to the submitted work. The other authors declare that they have no conflicts of interest.

## References

[1] Rajabiyazdi F., Alam R., Pal A. (2021). Understanding the meaning of recovery to patients undergoing abdominal surgery. JAMA Surg.

[2] Keny C., Dillon E., Russell M., Colley A., Yank V., Tang V. (2024). “It’s incapacitated me in so many ways”: older adults’ lived experience with postoperative symptoms at home after major elective surgery. Ann Surg.

[3] Sveinsdóttir H., Zoëga S., Ingadóttir B., Blöndal K. (2021). Symptoms of anxiety and depression in surgical patients at the hospital, 6 weeks and 6 months postsurgery: a questionnaire study. Nurs Open.

[4] Gandotra S., Daza J.F., Diep C. (2024). Psychological distress after inpatient noncardiac surgery: a secondary analysis of the measurement of exercise tolerance before surgery prospective cohort study. Ann Surg.

[5] O’Connor R.C., Kirtley O.J. (2018). The integrated motivational–volitional model of suicidal behaviour. Philos Trans R Soc Lond B Biol Sci.

[6] Noel C.W., Eskander A., Sutradhar R. (2021). Incidence of and factors associated with nonfatal self-injury after a cancer diagnosis in Ontario, Canada. JAMA Netw Open.

[7] Fernando S.M., Qureshi D., Sood M.M. (2021). Suicide and self-harm in adult survivors of critical illness: population based cohort study. BMJ.

[8] Nicholls S.G., Quach P., von Elm E. (2015). The REporting of Studies Conducted Using Observational Routinely-Collected Health Data (RECORD) statement: methods for arriving at consensus and developing reporting guidelines. PLoS One.

[9] Corbett C., Armstrong M.J., Parker R., Webb K., Neuberger J.M. (2013). Mental health disorders and solid-organ transplant recipients. Transplantation.

[10] O’Hara M.W., McCabe J.E. (2013). Postpartum depression: current status and future directions. Annu Rev Clin Psychol.

[11] Tully P.J. (2012). Psychological depression and cardiac surgery: a comprehensive review. J Extra Corpor Technol.

[12] Bethell J., Rhodes A.E. (2009). Identifying deliberate self-harm in emergency department data. Health Rep.

[13] Simon G.E., Shortreed S.M., Boggs J.M. (2022). Accuracy of ICD-10-CM encounter diagnoses from health records for identifying self-harm events. J Am Med Inform Assoc.

[14] Gatov E., Kurdyak P., Sinyor M., Holder L., Schaffer A. (2018). Comparison of vital statistics definitions of suicide against a coroner reference standard: a population-based linkage study. Can J Psychiatry.

[15] Stavem K., Hoel H., Skjaker S.A., Haagensen R. (2017). Charlson Comorbidity Index derived from chart review or administrative data: agreement and prediction of mortality in intensive care patients. Clin Epidemiol.

[16] Bekeris J., Wilson L.A., Fiasconaro M. (2020). New onset depression and anxiety after spinal fusion surgery: incidence and risk factors. Spine.

[17] Wilson B.R., Tringale K.R., Hirshman B.R. (2017). Depression after spinal surgery: a comparative analysis of the California Outcomes Database. Mayo Clin Proc.

[18] Funk M.J., Westreich D., Wiesen C., Stürmer T., Brookhart M.A., Davidian M. (2011). Doubly robust estimation of causal effects. Am J Epidemiol.

[19] Lau B., Cole S.R., Gange S.J. (2009). Competing risk regression models for epidemiologic data. Am J Epidemiol.

[20] Zhang Y., Hedo R., Rivera A., Rull R., Richardson S., Tu X.M. (2019). Post hoc power analysis: is it an informative and meaningful analysis?. Gen Psych.

[21] Potter A.L., Haridas C., Neumann K. (2023). Incidence, timing, and factors associated with suicide among patients undergoing surgery for cancer in the US. JAMA Oncol.

[22] Tidemalm D., Långström N., Lichtenstein P., Runeson B. (2008). Risk of suicide after suicide attempt according to coexisting psychiatric disorder: Swedish cohort study with long term follow-up. BMJ.

[23] Borges G., Nock M.K., Haro Abad J.M. (2010). Twelve-month prevalence of and risk factors for suicide attempts in the World Health Organization World Mental Health Surveys. J Clin Psychiatry.

[24] Crump C., Sundquist K., Sundquist J., Winkleby M.A. (2014). Sociodemographic, psychiatric and somatic risk factors for suicide: a Swedish national cohort study. Psychol Med.

[25] El-Gabalawy R., Sommer J.L., Pietrzak R. (2019). Post-traumatic stress in the postoperative period: current status and future directions. Can J Anesth/J Can Anesth.

[26] Zatzick D.F., Russo J.E., Katon W. (2003). Somatic, posttraumatic stress, and depressive symptoms among injured patients treated in trauma surgery. Psychosomatics.

[27] Chen Z., Luo R., Yang Y., Xiang Z. (2021). The prevalence of depression in degenerative spine disease patients: a systematic review and meta-analysis. Eur Spine J.

[28] Ye J., Shim R., Rust G. (2012). Health care avoidance among people with serious psychological distress: analyses of 2007 Health Information National Trends Survey. J Health Care Poor Underserved.

[29] Goldman-Mellor S., Olfson M., Lidon-Moyano C., Schoenbaum M. (2019). Association of suicide and other mortality with emergency department presentation. JAMA Netw Open.

[30] Swain R.S., Taylor L.G., Braver E.R., Liu W., Pinheiro S.P., Mosholder A.D. (2019). A systematic review of validated suicide outcome classification in observational studies. Int J Epidemiol.

