# Supplementary material for: Nonfatal self-injury in the first year after surgery: a population-based epidemiologic study
Source: Br J Anaesth. 2026 Jan 22;136(6):1935–41. doi: 10.1016/j.bja.2025.12.027 (PMC13197899; doi:10.1016/j.bja.2025.12.027)
Supplement: Multimedia component 1 [file mmc1.docx]

**Supplementary Appendix A.** Procedural codes used to identify eligible surgical procedures in the State Inpatient Database and State Ambulatory Surgery Database.

| **Surgical subspecialty** | **ICD-10-PCS** | **CPT** |
| --- | --- | --- |
| Breast | 0HTT, 0HTU, 0HTV, 0HTY, 0HBT, 0HBU, 0HBV, 0H5T, 0H5U, 0H5V | 19101, 19105, 19110, 19112, 19120, 19125, 19126, 19300, 19301, 19302, 19303, 19305, 19306, 19307, 19316, 19318, 19324, 19325, 19350, 19355, 19357, 19361, 19364, 19366, 19367, 19368, 19369 |
| ENT | Neck resection, excision, destruction:  0CTS, 0CBS, 0C5S, 0CDS | Neck and larynx dissection:  31300, 31360, 31365, 31367, 31368, 31370, 31375, 31380, 31382, 31390, 31395, 31400, 31420, 31551, 31552, 31553, 31554, 31560, 31561, 31580, 31584, 31587, 31590, 31591, 31592, 31599, 38308, 38542, 38720, 38724, 41135, 41140, 41145, 41155, 42420, 42425, 42426, 60000, 60280, 60281, 69150, 69155 |
|  | Thyroid and parathyroid:  0GTG, 0GTH, 0GTJ, 0GTK, 0GBG, 0GBH, 0GBJ, 0GBK, 0G5G, 0G5H, 0G5J, 0G5K, 0GTL, 0GTM, 0GTN, 0GTP, 0GTQ, 0GTR, 0GBL, 0GBM, 0GBN, 0GBP, 0GBQ, 0GBR, 0G5L, 0G5M, 0G5N, 0G5P, 0G5Q, 0G5R | Thyroid and parathyroid procedures:  60200, 60210, 60212, 60220, 60225, 60240, 60252, 60254, 60260, 60270, 60271, 60500, 60502, 60505, 60512 |
| General Surgery | Appendix:  0DTJ, 0DBJ, 0D5J | Appendix:  44900, 44950, 44955, 44960, 44970, 44979 |
|  | Liver:  0FT0, 0FT1, 0FT2, 0FT5, 0FT6, 0FT7, 0FT8, 0FT9, 0FTC, 0FB0, 0FB1, 0FB2, 0FB5, 0FB6, 0FB7, 0FB8, 0FB9, 0FBC, 0F50, 0F51, 0F52, 0F55, 0F56, 0F57, 0F58, 0F59, 0F5C, | Liver and bile duct:  47010, 47015, 47100, 47120, 47122, 47125, 47130, 47300, 47350, 47360, 47361, 47362, 47370, 47371, 47379, 47380, 47381, 47400, 47420, 47425, 47460, 47700, 47701, 47711, 47712, 47715, 47760, 47765, 47780, 47785, 47800, 47802, 47900 |
|  | Pancreas:  0FTD, 0FTF, 0FTG, 0FBD, 0FBF, 0FBG, 0F5D, 0F5F, 0F5G | Pancreas  48000, 48001, 48020, 48100, 48105, 48120, 48140, 48145, 48146, 48148, 48150, 48152, 48153, 48154, 48155, 48160, 48500, 48510, 48520, 48540, 48545, 48548 |
|  | Adrenal glands:  0GT3, 0GT4, 0GT5, 0GB3, 0GB4, 0GB5, 0G53, 0G54, 0G55 |  |
|  | Inguinal hernia:  0YQ5, 0YQ6, 0YQA | Inguinal hernia:  49505, 49507, 49520, 49521, 49525, 49550, 49553, 49555, 49557, 49650, 49651  Abdominal/parastomal hernia:  49591, 49592, 49593, 49594, 49595, 49596, 49613, 49614, 49615, 49616, 49617, 49618, 49621, 49622, 49659 |
|  | Spleen:  07TP | Spleen:  38100, 38101, 38102, 38115, 38120 |
|  | Gallbladder:  0FT4, 0FB4, 0F54 | Gallbladder:  47480, 47562, 47563, 47564, 47570, 47600, 47605, 47610, 47612, 47620, 47720, 47721, 47740, 47741 |
|  | Procedures on gastrointestinal tract (esophagus, stomach, small intestine, large intestine, sigmoid, anus, omentum, mesentery, peritoneum):  …resection:  0DT1, 0DT2, 0DT3, 0DT4, 0DT5, 0DT6, 0DT7, 0DT8, 0DT9, 0DTA, 0DTB, 0DTC, 0DTE, 0DTF, 0DTG, 0DTH, 0DTK, 0DTL, 0DTM, 0DTN, 0DTP, 0DTQ, 0DTR, 0DTU, 0DTV, 0DTW  …excision:  0DB10, 0DB13, 0DB14, 0DB1F, 0DB20, 0DB23, 0DB24, 0DB2F, 0DB30, 0DB33, 0DB34, 0DB3F, 0DB40, 0DB43, 0DB44, 0DB4F, 0DB50, 0DB53, 0DB54, 0DB5F, 0DB60, 0DB63, 0DB64, 0DB6F, 0DB70, 0DB73, 0DB74, 0DB7F, 0DB80, 0DB83, 0DB84, 0DB8F, 0DB90, 0DB93, 0DB94, 0DB9F, 0DBA0, 0DBA3, 0DBA4, 0DBAF, 0DBB0, 0DBB3, 0DBB4, 0DBBF, 0DBC0, 0DBC3, 0DBC4, 0DBCF, 0DBE0, 0DBE3, 0DBE4, 0DBEF, 0DBF0, 0DBF3, 0DBF4, 0DBFF, 0DBG0, 0DBG3, 0DBG4, 0DBGF, 0DBH0, 0DBH3, 0DBH4, 0DBHF, 0DBK0, 0DBK3, 0DBK4, 0DBKF, 0DBL0, 0DBL3, 0DBL4, 0DBLF, 0DBM0, 0DBM3, 0DBM4, 0DBMF, 0DBN0, 0DBN3, 0DBN4, 0DBNF, 0DBP0, 0DBP3, 0DBP4, 0DBPF, 0DBQ0, 0DBQ3, 0DBQ4, 0DBQF, 0DBR0, 0DBR3, 0DBR4, 0DBRF, 0DBU0, 0DBU3, 0DBU4, 0DBUF, 0DBV0, 0DBV3, 0DBV4, 0DBVF, 0DBW0, 0DBW3, 0DBW4, 0DBWF  …destruction:  0D510, 0D513, 0D514, 0D51F, 0D520, 0D523, 0D524, 0D52F, 0D530, 0D533, 0D534, 0D53F, 0D540, 0D543, 0D544, 0D54F, 0D550, 0D553, 0D554, 0D55F, 0D560, 0D563, 0D564, 0D56F, 0D570, 0D573, 0D574, 0D57F, 0D580, 0D583, 0D584, 0D58F, 0D590, 0D593, 0D594, 0D59F, 0D5A0, 0D5A3, 0D5A4, 0D5AF, 0D5B0, 0D5B3, 0D5B4, 0D5BF, 0D5C0, 0D5C3, 0D5C4, 0D5CF, 0D5E0, 0D5E3, 0D5E4, 0D5EF, 0D5F0, 0D5F3, 0D5F4, 0D5FF, 0D5G0, 0D5G3, 0D5G4, 0D5GF, 0D5H0, 0D5H3, 0D5H4, 0D5HF, 0D5K0, 0D5K3, 0D5K4, 0D5KF, 0D5L0, 0D5L3, 0D5L4, 0D5LF, 0D5M0, 0D5M3, 0D5M4, 0D5MF, 0D5N0, 0D5N3, 0D5N4, 0D5NF, 0D5P0, 0D5P3, 0D5P4, 0D5PF, 0D5Q0, 0D5Q3, 0D5Q4, 0D5QF, 0D5R0, 0D5R3, 0D5R4, 0D5RF, 0D5U0, 0D5U3, 0D5U4, 0D5UF, 0D5V0, 0D5V3, 0D5V4, 0D5VF, 0D5W0, 0D5W3, 0D5W4, 0D5WF  Gastric bypass:  0D16 | Gastric:  43360, 43605, 43620, 43621, 43622, 43631, 43632, 43633, 43634, 43640, 43641, 43644, 43645, 43770, 43771, 43772, 43773, 43774, 43775, 43800, 43810, 43820, 43825, 43840, 43842, 43843, 43845, 43847, 43848, 43860, 43865, 43870, 43880, 43886, 43887, 43888  Small bowel:  43496, 43846, 44010, 44020, 44021, 44120, 44125, 44126, 44127, 44130, 44186, 44187, 44202, 44300, 44310, 44312, 44314, 44316, 44602, 44603, 44615, 44640, 44650, 44800, 45136  Colon:  44025, 44110, 44111, 44137, 44140, 44141, 44143, 44144, 44145, 44146, 44147, 44150, 44151, 44155, 44156, 44157, 44158, 44160, 44188, 44204, 44205, 44206, 44207, 44208, 44210, 44211, 44212, 44213, 44227, 44320, 44322, 44340, 44345, 44346, 44604, 44605, 44620, 44625, 44626  Rectum:  45110, 45111, 45112, 45113, 45114, 45116, 45119, 45120, 45121, 45126, 45395, 45397, 45540, 45541, 45550, 45562, 45563, 57307 |
|  | Exploratory laparotomy:  20102, 35840, 39503, 39540, 39541, 43332, 43333, 44005, 44180, 44700, 44820, 44850, 49000, 49002, 49010, 49013, 49014, 49020, 49040, 49060, 49203, 49204, 49205, 49215, 49220, 49250, 49255, 49320, 49321, 49324, 49325, 49326, 49402, 49412, 49419, 49421, 49425, 49426, 49900, 49905, 49906, 58960 |  |
| Gynecology | Hysterectomy:  0UT9 | Hysterectomy:  58150, 58152, 58180, 58200, 58210, 58240, 58541, 58542, 58543, 58544, 58548, 58550, 58552, 58553, 58554, 58570, 58571, 58572, 58573, 58575, 58951, 58953, 58954, 58956, 51925, 58260, 58262, 58263, 58267, 58270, 58275, 58280, 58285, 58290, 58291, 58292, 58293, 58294 |
|  | Oophorectomy:  0UT0, 0UT1, 0UT2  Salpingectomy:  0UT5, 0UT6, 0UT7 | Procedures on ovaries:  58660, 58661, 58662, 58679, 58720, 58740, 58800, 58805, 58820, 58822, 58825, 58900, 58920, 58925, 58940, 58943, 58950, 58952 |
|  | Destruction/excision of female genital tract:  0U5, 0UB  Repair female genital tract:  0UQ0, 0UQ1, 0UQ2, 0UQ3, 0UQ4, 0UQ5, 0UQ6, 0UQ7, 0UQ9, 0UQC, 0UQF  Support female genital tract:  0UU0, 0UU1, 0UU2, 0UU3, 0UU4, 0UU5, 0UU6, 0UU7, 0UU9, 0UUC, 0UUF |  |
| Neurosurgery | Procedures on brain, meninges, ventricles, basal ganglia, thalamus, hypothalamus, pons, cerebellum, medulla oblongata, cranial nerves:  …destruction of:  0050, 0051, 0052, 005^, 0057, 0058, 0059, 005A, 005B, 005C, 005D, 005F, 005G, 005H, 005J, 005K, 005L, 005M, 005N, 005P, 005Q, 005R, 005S  …excision of:  00B0, 00B1, 00B2, 00B^, 00B7, 00B8, 00B9, 00BA, 00BB, 00BC, 00BD, 00BF, 00BG, 00BH, 00BJ, 00BK, 00BL, 00BM, 00BN, 00BP, 00BQ, 00BR, 00BS  …extraction of:  00D0, 00D1, 00D2, 00D^, 00D7, 00D8, 00D9, 00DA, 00DB, 00DC, 00DD, 00DF, 00DG, 00DH, 00DJ, 00DK, 00DL, 00DM, 00DN, 00DP, 00DQ, 00DR, 00DS  …release of:  00N0, 00N1, 00N2, 00N^, 00N7, 00N8, 00N9, 00NA, 00NB, 00NC, 00ND, 00NF, 00NG, 00NH, 00NJ, 00NK, 00NL, 00NM, 00NN, 00NP, 00NQ, 00NR, 00NS | Intracranial:  61105, 61107, 61108, 61120, 61140, 61150, 61151, 61154, 61156, 61210, 61250, 61253, 61304, 61305, 61312, 61313, 61314, 61315, 61320, 61321, 61322, 61323, 61330, 61333, 61340, 61343, 61345, 61458, 61460, 61510, 61512, 61514, 61516, 61518, 61519, 61520, 61521, 61522, 61524, 61526, 61530, 61531, 61533, 61534, 61535, 61536, 61537, 61538, 61539, 61540, 61541, 61543, 61544, 61545, 61546, 61548, 61566, 61567, 61570, 61571, 61575, 61576, 61580, 61581, 61582, 61583, 61584, 61585, 61586, 61590, 61591, 61592, 61595, 61598, 61600, 61601, 61605, 61606, 61607, 61608, 61615, 61616, 61618, 61619, 61680, 61682, 61684, 61686, 61690, 61692, 61697, 61698, 61700, 61702, 61703, 61705, 61708, 61710, 61711, 61720, 61735, 61736, 61737, 61750, 61751, 61760, 61770, 61850, 61860, 61863, 61867, 61870, 61880, 62000, 62005, 62010, 62100, 62120, 62121, 62161, 62163, 62164, 62165, 62200, 62201 |
|  |  | VP shunt:  62160, 62180, 62190, 62192, 62194, 62220, 62223, 62225, 62230, 62256, 62258 |
| Orthopedic | Lower limb…  …joint replacement:  0SR9, 0SRB, 0SRC, 0SRD, 0SRA, 0SRE, 0SRF, 0SRG, 0SRH, 0SRJ, 0SRK, 0SRL, 0SRM, 0SRN, 0SRP, 0SRQ, 0SRR, 0SRS, 0SRT, 0SRU, 0SRV, 0SRW  …joint fusion:  0SG9, 0SGB, 0SGC, 0SGD, 0SGF, 0SGG, 0SGH, 0SGJ, 0SGK, 0SGL, 0SGM, 0SGN, 0SGP, 0SGQ  …bone insertion:  0QH2, 0QH3, 0QH4, 0QH5, 0QH6, 0QH7, 0QH8, 0QH9, 0QHB, 0QHC, 0QHD, 0QHF, 0QHG, 0QHH, 0QHJ, 0QHK, 0QHL, 0QHM, 0QHN, 0QHP, 0QHQ | Hip prosthesis:  27125, 27130, 27132, 27134, 27137, 27138, 27236  Knee prosthesis:  27438, 27440, 27441, 27442, 27443, 27445, 27446, 27447, 27486, 27487  Lower limb open reduction of fracture:  27177, 27178, 27179, 27181, 27244, 27245, 27248, 27254, 27269, 27506, 27507, 27511, 27513, 27514, 27519, 27535, 27536, 27540, 27758, 27759, 27766, 27769, 27784, 27792, 27814, 27822, 27823, 27826, 27827, 27828, 27829 |
|  | Upper limb…  …joint replacement:  0RRE, 0RRF, 0RRG, 0RRH, 0RRJ, 0RRK, 0RRL, 0RRM, 0RRN, 0RRP, 0RRQ, 0RRR, 0RRS, 0RRT, 0RRU, 0RRV, 0RRW, 0RRX  …joint fusion:  0RGE, 0RGF, 0RGG, 0RGH, 0RGJ, 0RGK, 0RGL, 0RGM, 0RGN, 0RGP, 0RGQ, 0RGR, 0RGS, 0RGT, 0RGU, 0RGV, 0RGW, 0RGX  …bone insertion:  0PH5, 0PH6, 0PH7, 0PH8, 0PH9, 0PHB, 0PHC, 0PHD, 0PHF, 0PHG, 0PHH, 0PHJ, 0PHK, 0PHL, 0PHM, 0PHN, 0PHP, 0PHQ, 0PHR, 0PHS, 0PHT, 0PHV | Upper limb open reduction of fracture:  23615, 23616, 23630, 23670, 23680, 24515, 24516, 24545, 24546, 24575, 24579, 24586, 24587, 24635, 24665, 24666, 24685, 25337, 25515, 25525, 25526, 25545, 25574, 25575, 25607, 25608, 25609, 25652 |
|  | Amputation of lower limb:  0Y67, 0Y68, 0Y6C, 0Y6D, 0Y6F, 0Y6G, 0Y6H, 0Y6J, 0Y6M, 0Y6N, 0Y6P, 0Y6Q, 0Y6R, 0Y6S, 0Y6T, 0Y6U, 0Y6V, 0Y6W, 0Y6X, 0Y6Y | Amputation of…  …lower limb:  27290, 27295, 27590, 27591, 27592, 27598, 27880, 27881, 27882, 27884, 27886, 27888, 27889, 28124, 28126, 28160, 28800, 28805, 28810, 28820, 28825  …upper limb:  23900, 23920, 24900, 24920, 24930, 24931, 25900, 25905, 25909, 25920, 25922, 25924, 25927, 25929, 25931, 26235, 26236, 26910, 26951, 26952 |
| Spine | Decompression:  00NW, 00NX, 00NY  Fusion of vertebrae:  0RG0, 0RG1, 0RG2, 0RG4, 0RG6, 0RG7, 0RG8, 0RGA, 0SG0, 0SG1 | Fusion of vertebrae:  22532, 22533, 22548, 22551, 22554, 22556, 22558, 22586, 22590, 22595, 22600, 22610, 22612, 22630, 22633, 22800, 22802, 22804, 22808, 22810, 22812, 27280 |
|  | Destruction of disc:  0R53, 0R55, 0R59, 0R5B, 0S52, 0S54  Excision of disc:  0RB3, 0RB5, 0RB9, 0RBB, 0SB2, 0SB4  Resection of disc:  0RT3, 0RT5, 0RT9, 0RTB, 0ST2, 0ST4 | Laminectomy:  22220, 22222, 22224, 22856, 22857, 22861, 22862, 22867, 62287, 62351, 62380, 63001, 63003, 63005, 63011, 63012, 63015, 63016, 63017, 63020, 63030, 63035, 63040, 63042, 63045, 63046, 63047, 63048, 63050, 63051, 63055, 63056, 63064, 63075, 63077, 63081, 63082, 63085, 63086, 63087, 63088, 63090, 63091, 63101, 63102, 63103, 63170, 63172, 63173, 63180, 63182, 63185, 63190, 63191, 63197, 63200, 63250, 63251, 63252, 63265, 63266, 63267, 63268, 63270, 63271, 63272, 63273, 63275, 63276, 63277, 63278, 63280, 63281, 63282, 63283, 63285, 63286, 63287, 63290, 63709, 63740, 0202T, 0219T, 0220T, 0221T |
| Thoracic | Procedures on respiratory system (trachea, carina, main bronchi, lobes:  …resection:  0BT  …excision:  0BB10, 0BB13, 0BB14, 0BB20, 0BB23, 0BB24, 0BB30, 0BB33, 0BB34, 0BB40, 0BB43, 0BB44, 0BB50, 0BB53, 0BB54, 0BB60, 0BB63, 0BB64, 0BB70, 0BB73, 0BB74, 0BB80, 0BB83, 0BB84, 0BB90, 0BB93, 0BB94, 0BBB0, 0BBB3, 0BBB4, 0BBC0, 0BBC3, 0BBC4, 0BBD0, 0BBD3, 0BBD4, 0BBF0, 0BBF3, 0BBF4, 0BBG0, 0BBG3, 0BBG4, 0BBH0, 0BBH3, 0BBH4, 0BBJ0, 0BBJ3, 0BBJ4, 0BBK0, 0BBK3, 0BBK4, 0BBL0, 0BBL3, 0BBL4, 0BBM0, 0BBM3, 0BBM4, 0BBN0, 0BBN3, 0BBN4,  Procedures on pleura or diaphragm:  0BBP0, 0BBP3, 0BBP4, 0BBT0, 0BBT3, 0BBT4 | Lung, pleura, diaphragm:  20101, 21603, 31770, 31775, 32096, 32097, 32100, 32110, 32120, 32124, 32140, 32141, 32151, 32200, 32215, 32220, 32225, 32310, 32320, 32440, 32442, 32445, 32480, 32482, 32484, 32486, 32488, 32491, 32501, 32503, 32504, 32505, 32506, 32507, 32540, 32553, 32607, 32608, 32609, 32651, 32652, 32655, 32662, 32663, 32666, 32667, 32668, 32669, 32670, 32671, 32672, 32800, 32815, 32905, 32906, 32940, 32960, 39000, 39010, 39200, 39220, 39501, 39545, 39560, 39561, 64746 |
|  |  | Thoracic approach to esophagus:  43117, 43118, 43121, 43122, 43286, 43287, 43288, 43320 |
| Urology | Kidney:  0T50, 0T51, 0T53, 0T54, 0T55, 0T56, 0T5B, 0T5C, 0T5D, 0TB0, 0TB1, 0TB3, 0TB4, 0TB5, 0TB6, 0TBB, 0TBC, 0TBD, 0TT0, 0TT1, 0TT3, 0TT4, 0TT5, 0TT6, 0TTB, 0TTC, 0TTD, 0TD | Kidney:  50010, 50020, 50040, 50045, 50060, 50065, 50070, 50075, 50120, 50125, 50130, 50135, 50205, 50220, 50225, 50230, 50234, 50236, 50240, 50250, 50280, 50290, 50320, 50400, 50405, 50541, 50542, 50543, 50545, 50546, 50547, 50548, 50549 |
|  | Prostate:  0VT0 | Prostate:  55705, 55810, 55812, 55815, 55821, 55831, 55840, 55842, 55845, 55866, 55867 |
| Vascular | Carotid endarterectomy:  03CH, 03CJ, 03CK, 03CL, 03CM, 03CN | Carotid endarterectomy:  35301, 35390 |
|  | Abdominal aortic aneurysm repair:  04V0 | Abdominal aortic aneurysm repair:  34830, 34831, 34832, 35081, 35082, 35091, 35092, 35102, 35103 |
|  | Peripheral bypass:  0410, 041C, 041D, 041E, 041F, 041H, 041J, 041K, 041L, 041M, 041N | Peripheral bypass:  33889, 35501, 35506, 35508, 35509, 35510, 35511, 35512, 35515, 35516, 35518, 35521, 35522, 35523, 35525, 35533, 35556, 35558, 35566, 35570, 35571, 35583, 35585, 35587, 35616, 35621, 35623, 35650, 35654, 35656, 35661, 35666, 35671, 35686 |

**Supplementary Appendix B.** ICD-10-CM codes for identifying emergency department visits or hospitalizations due to nonfatal self-injury.

| **ICD-10-CM Codes** | **Description** |
| --- | --- |
|  | *Intentional self-poisoning (or poisoning of undetermined intent) by…* |
| X60 (Y10) | nonopioid analgesics, antipyretics and antirheumatics |
| X61 (Y11) | antiepileptic, sedative-hypnotic, antiparkinsonism and psychotropic drugs, not elsewhere classified |
| X62 (Y12) | narcotics and psychodysleptics (hallucinogens), not elsewhere classified |
| X63 (Y13) | other drugs acting on the autonomic nervous system |
| X64 (Y14) | other and unspecified drugs, medicaments and biological substances |
| X65 (Y15) | alcohol |
| X66 (Y16) | organic solvents and halogenated hydrocarbons and their vapours |
| X67 (Y17) | carbon monoxide and other gases and vapours |
| X68 (Y18) | pesticides |
| X69 (Y19) | other and unspecified chemicals and noxious substances |
|  |  |
|  | *Poisoning with self-harm intent by…* |
| T36..2 | systemic antibiotics |
| T37..2 | other systemic anti-infectives and antiparasitics |
| T38..2 | hormones and their synthetic substitutes and antagonists, not elsewhere classified |
| T39..2 | nonopioid analgesics, antipyretics and antirheumatics |
| T40..2 | narcotics and psychodysleptics (hallucinogens) |
| T41..2 | anaesthetics and therapeutic gases |
| T42..2 | antiepileptic, sedative-hypnotic and antiparkinsonism drugs |
| T43..2 | psychotropic drugs, not elsewhere classified |
| T44..2 | drugs primarily affecting the autonomic nervous system |
| T45..2 | primarily systemic and haematological agents, not elsewhere classified |
| T46..2 | agents primarily affecting the cardiovascular system |
| T47..2 | agents primarily affecting the gastrointestinal system |
| T48..2 | agents primarily acting on smooth and skeletal muscles and the respiratory system |
| T49..2 | topical agents primarily affecting skin and mucous membrane and by ophthalmological, otorhinolaryngological and dental drugs |
| T50..2 | diuretics and other and unspecified drugs, medicaments and biological substances |
| T51..2 | alcohol |
| T52..2 | organic solvents |
| T53..2 | halogen derivatives of aliphatic and aromatic hydrocarbons |
| T54..2 | corrosive substances |
| T55..2 | soaps and detergents |
| T56..2 | metals |
| T57..2 | other inorganic substances |
| T58..2 | carbon monoxide |
| T59..2 | other gases, fumes and vapours |
| T60..2 | Toxic effect of pesticides |
| T61..2 | noxious substances eaten as seafood |
| T62..2 | other noxious substances eaten as food |
| T63..2 | contact with venomous animals |
| T64..2 | aflatoxin and other mycotoxin food contaminants |
| T65..2 | other and unspecified substances |
|  |  |
|  | *Intentional self-harm (or harm of undetermined intent) by…* |
| X70 (Y20) | hanging, strangulation and suffocation |
| X71 (Y21) | drowning and submersion |
| X72 (Y22) | handgun discharge |
| X73 (Y23) | rifle, shotgun and larger firearm discharge |
| X74 (Y24) | other and unspecified firearm discharge |
| X75 (Y25) | explosive material |
| X76 (Y26) | smoke, fire and flames |
| X77 (Y27) | steam, hot vapours and hot objects |
| X78 (Y28) | sharp object |
| X79 (Y29) | blunt object |
| X80 (Y30) | jumping from a high place |
| X81 (Y31) | jumping or lying before moving object |
| X82 (Y32) | crashing of motor vehicle |
| X83 (Y33) | other specified means |
| X84 (Y34) | unspecified means |
|  |  |
|  | *Miscellaneous* |
| T71..2 | Asphyxiation |
| Y870, Y872 | Sequelae of intentional self-harm (or events of undetermined intent) |
| R4588 | Non-suicidal self-harm |
| T1491 | Suicide attempt (without further details) |

Codes in parentheses represent equivalent causes as described but with undetermined intent.

“.” in codes represent filler positions which can be any character or digit value.

**Supplementary Appendix C.** ICD-10-CM codes for prior psychiatric diagnoses.

| **ICD-10-CM Codes** | **Description** |
| --- | --- |
|  | Neurotic, stress-related and somatoform disorders |
| F40 | Phobic anxiety disorders |
| F41 | Other anxiety disorders |
| F42 | Obsessive-compulsive disorder |
| F43 | Reaction to severe stress, and adjustment disorders |
| F44 | Dissociative (conversion) disorders |
| F45 | Somatoform disorders |
| F48 | Other neurotic disorders |
|  |  |
|  | Mood (affective) disorders |
| F30 | Manic episode |
| F31 | Bipolar affective disorder |
| F32 | Depressive episode |
| F33 | Recurrent depressive disorder |
| F34 | Persistent mood (affective) disorders |
| F38 | Other mood (affective) disorders |
| F39 | Unspecified mood (affective) disorder |
|  |  |
|  | Schizophrenia, schizotypal and delusional disorders |
| F20 | Schizophrenia |
| F21 | Schizotypal disorder |
| F22 | Persistent delusional disorders |
| F23 | Acute and transient psychotic disorders |
| F24 | Induced delusional disorder |
| F25 | Schizoaffective disorders |
| F28 | Other nonorganic psychotic disorders |
| F29 | Unspecified nonorganic psychosis |
|  |  |
|  | Mental and behavioural disorders due to psychoactive substance use |
| F10 | Mental and behavioural disorders due to use of alcohol |
| F11 | Mental and behavioural disorders due to use of opioids |
| F12 | Mental and behavioural disorders due to use of cannabinoids |
| F13 | Mental and behavioural disorders due to use of sedatives or hypnotics |
| F14 | Mental and behavioural disorders due to use of cocaine |
| F15 | Mental and behavioural disorders due to use of other stimulants, including caffeine |
| F16 | Mental and behavioural disorders due to use of hallucinogens |
| F17 | Mental and behavioural disorders due to use of tobacco |
| F18 | Mental and behavioural disorders due to use of volatile solvents |
| F19 | Mental and behavioural disorders due to multiple drug use and use of other psychoactive substances |

**Supplementary Appendix D.** Procedural codes used to identify percutaneous coronary intervention (PCI) in the State Inpatient Database and State Ambulatory Surgery Database.

| **ICD-10-PCS** | **CPT** |
| --- | --- |
| 0270, 0271, 0272, 0273 | 92920, 92921, 92922, 92923, 92924, 92925, 92926, 92927, 92928, 92929, 92930, 92931, 92932, 92933, 92934, 92935, 92936, 92937, 92938, 92939, 92940, 92941, 92942, 92943, 92944 |

**Supplementary Appendix E.** Procedural codes used to identify cataract procedures in the State Inpatient Database and State Ambulatory Surgery Database.

| **ICD-10-PCS** | **CPT** |
| --- | --- |
| 08DJ3ZZ, 08DK3ZZ, 08RJ3JZ, 08RK3JZ | 66820, 66821, 66920, 66940, 66982, 66983, 66984 |

**Supplementary Appendix F.** Causes of 824 index non-fatal self-injuries at emergency department visit or hospital admission**.**

| **Description** | **ICD-10-CM Code** | **Frequency** | **% of cases** |
| --- | --- | --- | --- |
| Poisoning with self-harm intent by antiepileptic, sedative-hypnotic and antiparkinsonism drugs | T42..2 | 134 | 16.3 |
| Poisoning with self-harm intent by psychotropic drugs, not elsewhere classified | T43..2 | 91 | 11.0 |
| Suicide attempt (without further details) | T1491 | 85 | 10.3 |
| Intentional self-harm by sharp object | X78 | 82 | 10.0 |
| Harm of undetermined intent by other specified means | Y33 | 68 | 8.3 |
| Poisoning with self-harm intent by nonopioid analgesics, antipyretics and antirheumatics | T39..2 | 66 | 8.0 |
| Poisoning with self-harm intent by narcotics and psychodysleptics (hallucinogens) | T40..2 | 66 | 8.0 |
| Poisoning with self-harm intent by diuretics and other and unspecified drugs, medicaments and biological substances | T50..2 | 61 | 7.4 |
| Intentional self-harm by other specified means | X83 | 31 | 3.8 |
| Harm of undetermined intent by sharp object | Y28 | 25 | 3.0 |
| Poisoning with self-harm intent by primarily systemic and haematological agents, not elsewhere classified | T45..2 | 17 | 2.1 |
| Poisoning with self-harm intent by agents primarily affecting the cardiovascular system | T46..2 | 13 | 1.6 |
| Harm of undetermined intent by blunt object | Y29 | 12 | 1.5 |
| Poisoning with self-harm intent by agents primarily acting on smooth and skeletal muscles and the respiratory system | T48..2 | 12 | 1.5 |
| Poisoning with self-harm intent by hormones and their synthetic substitutes and antagonists, not elsewhere classified | T38..2 | 10 | 1.2 |
| Poisoning with self-harm intent by drugs primarily affecting the autonomic nervous system | T44..2 | 10 | 1.2 |
| Poisoning with self-harm intent by alcohol | T51..2 | 6 | 0.7 |
| Harm of undetermined intent by other and unspecified firearm discharge | Y24 | 4 | 0.5 |
| Harm of undetermined intent by jumping from a high place | Y30 | 4 | 0.5 |
| Poisoning with self-harm intent by other and unspecified substances | T65..2 | 4 | 0.5 |
| Harm of undetermined intent by steam, hot vapours and hot objects | Y27 | 3 | 0.4 |
| Poisoning with self-harm intent by asphyxiation | T71..2 | 3 | 0.4 |
| Intentional self-harm by blunt object | X79 | 2 | 0.2 |
| Harm of undetermined intent by smoke, fire and flames | Y26 | 2 | 0.2 |
| Poisoning with self-harm intent by organic solvents | T52..2 | 2 | 0.2 |
| Poisoning with self-harm intent by corrosive substances | T54..2 | 2 | 0.2 |
| Poisoning with self-harm intent by metals | T56..2 | 2 | 0.2 |
| Intentional self-harm by other and unspecified firearm discharge | X74 | 1 | 0.1 |
| Intentional self-harm by jumping or lying before moving object | X81 | 1 | 0.1 |
| Intentional self-harm by crashing of motor vehicle | X82 | 1 | 0.1 |
| Harm of undetermined intent by handgun discharge | Y22 | 1 | 0.1 |
| Poisoning with self-harm intent by other systemic anti-infectives and antiparasitics | T37..2 | 1 | 0.1 |
| Poisoning with self-harm intent by agents primarily affecting the gastrointestinal system | T47..2 | 1 | 0.1 |
| Poisoning with self-harm intent by soaps and detergents | T55..2 | 1 | 0.1 |

“.” in codes represent filler positions which can be any character or digit value.

**Supplementary Appendix G.** Counts of NFSI in the first year after surgery.

| **No. of NFSI events** | **No. of unique patients** |
| --- | --- |
| 1 | 755 |
| 2 | 60 |
| 3 | 6 |
| 4 | 2 |
| 5 | 0 |
| 6 | 0 |
| 7 | 1 |

**Supplementary Appendix H.** Characteristics of combined cohort of patients in the primary and comparator (percutaneous coronary intervention) cohorts, before and after 1:1 matching.

|  | **Before matching** | | | **After matching** | | |
| --- | --- | --- | --- | --- | --- | --- |
|  | **PCI n=84,082** | **Surgery n=1,157,974** | **SMD** | **PCI n=82,798** | **Surgery  n=82,798** | **SMD** |
| **Age (years)** |  |  | 0.657 |  |  | 0.198 |
| 18–39 | 43,338 (51.5%) | 387,821 (33.5%) |  | 42,792 (51.7%) | 39,132 (47.3%) |  |
| 40–64 | 39,355 (46.8%) | 537,171 (46.4%) |  | 38,633 (46.7%) | 39,458 (47.7%) |  |
| 65+ | 1,389 (1.7%) | 232,982 (20.1%) |  | 1,373 (1.7%) | 4,208 (5.1%) |  |
| **Female sex** | 25,089 (29.8%) | 724,282 (62.5%) | 0.695 | 24,824 (30.0%) | 26,715 (32.3%) | 0.049 |
| **Race** |  |  | 0.187 |  |  | 0.106 |
| White | 18,175 (21.6%) | 172,677 (14.9%) |  | 17,713 (21.4%) | 18,095 (21.9%) |  |
| Black | 7,590 (9.0%) | 126,170 (10.9%) |  | 7,527 (9.1%) | 9,038 (10.9%) |  |
| Hispanic | 7,370 (8.8%) | 128,133 (11.1%) |  | 7,301 (8.8%) | 9,036 (10.9%) |  |
| Other | 50,947 (60.6%) | 730,994 (63.1%) |  | 50,257 (60.7%) | 46,629 (56.3%) |  |
| **Income (quartile)** |  |  | 0.019 |  |  | 0.105 |
| 4 (highest) | 21,945 (26.3%) | 309,606 (26.9%) |  | 21,814 (26.3%) | 19,998 (24.2%) |  |
| 3 | 22,557 (27.1%) | 302,277 (26.3%) |  | 22,358 (27.0%) | 24,055 (29.1%) |  |
| 2 | 20,584 (24.7%) | 283,215 (24.6%) |  | 20,433 (24.7%) | 18,022 (21.8%) |  |
| 1 (lowest) | 18,270 (21.9%) | 253,922 (22.1%) |  | 18,193 (22.0%) | 20,723 (25.0%) |  |
| **Health insurance** |  |  | 0.604 |  |  | 0.238 |
| Private | 27,060 (32.2%) | 514,017 (44.4%) |  | 26,551 (32.1%) | 20,353 (24.6%) |  |
| Medicare | 40,848 (48.6%) | 378,135 (32.7%) |  | 40,443 (48.8%) | 39,305 (47.5%) |  |
| Medicaid | 13,266 (15.8%) | 198,205 (17.1%) |  | 13,065 (15.8%) | 18,163 (21.9%) |  |
| Uninsured/Other | 2,803 (3.3%) | 66,927 (5.8%) |  | 2,739 (3.3%) | 4,977 (6.0%) |  |
| **Rural location** | 6,271 (7.5%) | 97,491 (8.4%) | 0.035 | 6,072 (7.3%) | 6,660 (8.0%) | 0.027 |
| **Charlson Comorbidity** |  |  | 1.042 |  |  | 0.106 |
| None (0 points) | 11,502 (13.7%) | 654,264 (56.5%) |  | 11,329 (13.7%) | 13,445 (16.2%) |  |
| Mild (1–2) | 50,859 (60.5%) | 420,591 (36.3%) |  | 49,969 (60.4%) | 45,766 (55.3%) |  |
| Moderate/Severe (3+) | 21,721 (25.8%) | 83,209 (7.2%) |  | 21,500 (26.0%) | 23,587 (28.5%) |  |
| **Anxiety disorder** | 6,286 (7.5%) | 113,517 (9.8%) | 0.083 | 6,226 (7.5%) | 11,990 (14.5%) | 0.224 |
| **Mood disorder** | 5,593 (6.7%) | 96,695 (8.4%) | 0.065 | 5,534 (6.7%) | 13,260 (16.0%) | 0.297 |
| **Psychotic disorder** | 356 (0.4%) | 5,075 (0.4%) | 0.002 | 355 (0.4%) | 1,434 (1.7%) | 0.093 |
| **Substance use disorder** | 17,691 (21.0%) | 122,562 (10.6%) | 0.294 | 17,223 (20.8%) | 23,728 (28.7%) | 0.097 |
| **History of suicidality** | 74 (0.1%) | 876 (0.1%) | 0.01 | 73 (0.1%) | 304 (0.4%) | 0.059 |
| **Non-elective surgery** | 65,125 (77.6%) | 241,013 (20.8%) | 1.381 | 64,281 (77.6%) | 67,520 (81.5%) | 0.097 |

PCI: percutaneous coronary intervention, SMD: standardized mean difference.

All values are presented as n (%).

**Supplementary Appendix I.** Multivariable Cox model of nonfatal self-injury in the first year after surgery for patients undergoing surgery compared to patients undergoing percutaneous coronary intervention.

|  | Adjusted HR (95% CI) |
| --- | --- |
| **Surgery vs. PCI** | **1.55 (1.06–2.27)** |
| **Age (years)** |  |
| 18–39 | **4.96 (2.46–9.99)** |
| 40–64 | **2.45 (1.48–4.07)** |
| 65+ | 1 (ref) |
| **Race** |  |
| White | **2.09 (1.23–3.56)** |
| Black | 0.79 (0.36–1.73) |
| Hispanic | 1.88 (0.97–3.63) |
| Other | 1 (ref) |
| **Income (quartile)** |  |
| 1^st^ (lowest) | 1.03 (0.60–1.77) |
| 2^nd^ | 1.18 (0.70–2.00) |
| 3^rd^ | 1.32 (0.79–2.21) |
| 4^th^ (highest) | 1 (ref) |
| **Health insurance** |  |
| Private | 1 (ref) |
| Medicare | **3.30 (1.75–6.24)** |
| Medicaid | **4.88 (2.73–8.72)** |
| Uninsured/Other | 1.94 (0.75–5.05) |
| **Charlson Comorbidity** |  |
| None (0 points) | 1 (ref) |
| Mild (1–2) | 1.09 (0.68–1.75) |
| Moderate/Severe (3+) | 0.73 (0.41–1.29) |
| **Anxiety disorder** | **1.75 (1.17–2.62)** |
| **Mood disorder** | **2.16 (1.45–3.20)** |
| **Psychotic disorder** | **3.58 (1.85–6.89)** |
| **Substance use disorder** | **1.97 (1.39–2.80)** |

PCI: percutaneous coronary intervention.

Bolded estimates are statistically significant at p<0.05.

**Supplementary Appendix J.** Characteristics of combined cohort of patients in the primary and comparator (cataract procedures) cohorts, before and after 1:1 matching.

|  | **Before matching** | | | **After matching** | | |
| --- | --- | --- | --- | --- | --- | --- |
|  | **Cataract n=367,302** | **Surgery n=1,138172** | **SMD** | **Cataract n=233,307** | **Surgery n=233,307** | **SMD** |
| **Age (years)** |  |  | 1.068 |  |  | 0.130 |
| 18–39 | 280,117 (76.3%) | 370,653 (32.6%) |  | 147,177 (63.1%) | 159,431 (68.3%) |  |
| 40–64 | 85,002 (23.1%) | 534,542 (47.0%) |  | 83,995 (36.0%) | 70,461 (30.2%) |  |
| 65+ | 2,183 (0.6%) | 232,977 (20.5%) |  | 2,135 (0.9%) | 3,415 (1.5%) |  |
| **Female sex** | 217,809 (59.3%) | 710,468 (62.4%) | 0.064 | 133,335 (57.2%) | 128,132 (54.9%) | 0.045 |
| **Race** |  |  | 0.459 |  |  | 0.051 |
| White | 125,131 (34.1%) | 170,522 (15.0%) |  | 54,389 (23.3%) | 58,528 (25.1%) |  |
| Black | 26,593 (7.2%) | 124,841 (11.0%) |  | 18,119 (7.8%) | 19,582 (8.4%) |  |
| Hispanic | 30,100 (8.2%) | 126,408 (11.1%) |  | 19,331 (8.3%) | 18,833 (8.1%) |  |
| Other | 185,475 (50.5%) | 716,401 (62.9%) |  | 141,468 (60.6%) | 136,364 (58.4%) |  |
| **Income (quartile)** |  |  | 0.028 |  |  | 0.088 |
| 4 (highest) | 97,860 (26.8%) | 304,888 (27.0%) |  | 67,415 (28.9%) | 75,997 (32.6%) |  |
| 3 | 98,369 (27.0%) | 296,781 (26.3%) |  | 61,494 (26.4%) | 55,941 (24.0%) |  |
| 2 | 91,603 (25.1%) | 277,962 (24.6%) |  | 57,551 (24.7%) | 57,926 (24.8%) |  |
| 1 (lowest) | 76,856 (21.1%) | 249,781 (22.1%) |  | 46,847 (20.1%) | 43,443 (18.6%) |  |
| **Health insurance** |  |  | 0.596 |  |  | 0.200 |
| Private | 107,782 (29.5%) | 510,774 (44.9%) |  | 70,848 (30.4%) | 56,971 (24.4%) |  |
| Medicare | 217,546 (59.6%) | 362,853 (31.9%) |  | 132,646 (56.9%) | 133,759 (57.3%) |  |
| Medicaid | 25,776 (7.1%) | 197,433 (17.4%) |  | 20,093 (8.6%) | 24,038 (10.3%) |  |
| Uninsured/Other | 13,987 (3.8%) | 66,437 (5.8%) |  | 9,720 (4.2%) | 18,539 (7.9%) |  |
| **Rural location** | 31,041 (8.5%) | 95,434 (8.4%) | 0.002 | 21,218 (9.1%) | 24,813 (10.6%) | 0.052 |
| **Charlson Comorbidity** |  |  | 0.732 |  |  | 0.128 |
| None (0 points) | 320,114 (87.2%) | 645,090 (56.7%) |  | 186,693 (80.0%) | 176,858 (75.8%) |  |
| Mild (1–2%) | 44,222 (12.0%) | 411,082 (36.1%) |  | 43,679 (18.7%) | 50,245 (21.5%) |  |
| Moderate/Severe (3+) | 2,966 (0.8%) | 82,000 (7.2%) |  | 2,935 (1.3%) | 6,204 (2.7%) |  |
| **Anxiety disorder** | 5,791 (1.6%) | 111,357 (9.8%) | 0.36 | 5,695 (2.4%) | 10,849 (4.7%) | 0.12 |
| **Mood disorder** | 4,633 (1.3%) | 94,647 (8.3%) | 0.335 | 4,547 (1.9%) | 10,232 (4.4%) | 0.139 |
| **Psychotic disorder** | 316 (0.1%) | 5,501 (0.4%) | 0.100 | 296 (0.1%) | 1,014 (0.4%) | 0.058 |
| **Substance use disorder** | 6,078 (1.7%) | 121,140 (10.7%) | 0.383 | 5,961 (2.6%) | 12,857 (5.5%) | 0.154 |
| **History of suicidality** | 6 (0.0%) | 860 (0.1%) | 0.038 | 6 (0.0%) | 151 (0.1%) | 0.034 |
| **Non-elective surgery** | 148 (0.0%) | 237,671 (20.9%) | 0.724 | 148 (0.1%) | 2,418 (1.0%) | 0.132 |

SMD: standardized mean difference.

All values are presented as n (%).

**Supplementary Appendix K.** Multivariable Cox model of nonfatal self-injury in the first year after surgery for patients undergoing surgery compared to patients undergoing cataract procedures.

|  | Adjusted HR (95% CI) |
| --- | --- |
| **Surgery vs. cataract** | **1.48 (1.04–2.09)** |
| **Age (years)** |  |
| 18–39 | **8.16 (3.39–19.60)** |
| 40–64 | **3.12 (2.01–4.86)** |
| 65+ | 1 (ref) |
| **Health insurance** |  |
| Private | 1 (ref) |
| Medicare | **2.64 (1.56–4.44)** |
| Medicaid | **4.16 (2.55–6.77)** |
| Uninsured/Other | 1.18 (0.51–2.74) |
| **Charlson Comorbidity** |  |
| None (0 points) | 1 (ref) |
| Mild (1–2) | **1.65 (1.16–2.35)** |
| Moderate/Severe (3+) | 0.58 (0.18–1.88) |
| **Anxiety disorder** | 1.06 (0.62–1.83) |
| **Mood disorder** | **4.38 (2.77–6.94)** |
| **Substance use disorder** | **1.86 (1.17–2.94)** |
| **Non-elective surgery** | 1.05 (0.40–2.78) |

Bolded estimates are statistically significant at p<0.05.

**Supplementary Appendix L.** Proportion of sample without NFSI after surgery, stratified by age group.

**Supplementary Appendix M.** Proportion of sample without NFSI after surgery, stratified by sex.

**Supplementary Appendix N.** Proportion of sample without NFSI after surgery, stratified by race.

**Supplementary Appendix O.** Proportion of sample without NFSI after surgery, stratified by income quartile.

**Supplementary Appendix P.** Proportion of sample without NFSI after surgery, stratified by type of health insurance.

**Supplementary Appendix Q.** Proportion of sample without NFSI after surgery, stratified by location.

**Supplementary Appendix R.** Proportion of sample without NFSI after surgery, stratified by Charlson Comorbidity Index.

**Supplementary Appendix S.** Proportion of sample without NFSI after surgery, stratified by prior anxiety disorder diagnosis.

**Supplementary Appendix T.** Proportion of sample without NFSI after surgery, stratified by prior mood disorder diagnosis.

**Supplementary Appendix U.** Proportion of sample without NFSI after surgery, stratified by prior psychotic disorder diagnosis.

**Supplementary Appendix V.** Proportion of sample without NFSI after surgery, stratified by prior substance use disorder diagnosis.

**Supplementary Appendix W.** Proportion of sample without NFSI after surgery, stratified by prior history of suicidal ideation or nonfatal self-injury.

**Supplementary Appendix X.** Proportion of sample without NFSI after surgery, stratified by urgency of surgical procedure.

**Supplementary Appendix Y.** Proportion of sample without NFSI after surgery, stratified by subspecialty of surgical procedure.

**Supplementary Appendix Z.** Associations between predictor variables and nonfatal self-injury in the first year after surgery from Fine and Gray models accounting for the competing risk of death.

|  | Rate per 10,000 | Crude HR (95% CI) | Adjusted HR (95% CI) |
| --- | --- | --- | --- |
| Overall sample | 7.08 |  |  |
| **Age (years)** |  |  |  |
| 18–39 | 15.62 | **6.95 (5.51–8.78)** | **9.14 (6.65–12.55)** |
| 40–64 | 6.72 | **2.97 (2.35–3.74)** | **3.86 (2.89–5.16)** |
| 65+ | 2.33 | 1 (ref) | 1 (ref) |
| **Sex** |  |  |  |
| Female | 7.86 | **1.38 (1.19–1.61)** | **1.19 (1.01–1.40)** |
| Male | 5.76 | 1 (ref) | 1 (ref) |
| **Race** |  |  |  |
| White | 7.72 | **1.83 (1.43–2.34)** | **2.00 (1.55–2.58)** |
| Black | 6.93 | **1.63 (1.19–2.23)** | 1.31 (0.96–1.80) |
| Hispanic | 7.36 | **1.75 (1.28–2.38)** | 1.25 (0.92–1.71) |
| Other | 4.22 | 1 (ref) | 1 (ref) |
| **Income (quartile)** |  |  |  |
| 1^st^ (lowest) | 9.92 | **2.54 (2.05–3.17)** | **1.43 (1.13–1.81)** |
| 2^nd^ | 8.23 | **2.17 (1.74–2.7)** | **1.36 (1.08–1.72)** |
| 3^rd^ | 6.72 | **1.73 (1.38–2.17)** | **1.38 (1.10–1.73)** |
| 4^th^ (highest) | 3.88 | 1 (ref) | 1 (ref) |
| **Health insurance** |  |  |  |
| Private | 4.66 | 1 (ref) | 1 (ref) |
| Medicare | 4.67 | 0.97 (0.80–1.18) | **2.35 (1.84–3.01)** |
| Medicaid | 17.70 | **3.78 (3.20–4.45)** | **2.78 (2.33–3.33)** |
| Uninsured/Other | 7.65 | **1.64 (1.21–2.22)** | **1.53 (1.12–2.09)** |
| **Patient location** |  |  |  |
| Urban | 6.51 | 1 (ref) | 1 (ref) |
| Rural | 13.14 | **2.02 (1.67–2.45)** | **1.39 (1.13–1.70)** |
| **Charlson Comorbidity** |  |  |  |
| None (0 points) | 6.90 | 1 (ref) | 1 (ref) |
| Mild (1–2) | 7.70 | 1.09 (0.95–1.26) | **1.19 (1.02–1.39)** |
| Moderate/Severe (3+) | 5.26 | **0.68 (0.49–0.94)** | 0.88 (0.62–1.25) |
| **Anxiety disorder** |  |  |  |
| No | 5.74 | 1 (ref) | 1 (ref) |
| Yes | 19.36 | **3.37 (2.88–3.94)** | **1.76 (1.46–2.11)** |
| **Mood disorder** |  |  |  |
| No | 5.50 | 1 (ref) | 1 (ref) |
| Yes | 24.45 | **4.43 (3.81–5.16)** | **2.46 (2.05–2.95)** |
| **Psychotic disorder** |  |  |  |
| No | 6.89 | 1 (ref) | 1 (ref) |
| Yes | 52.15 | **7.26 (4.88–10.82)** | **2.94 (1.93–4.47)** |
| **Substance use disorder** |  |  |  |
| No | 5.50 | 1 (ref) | 1 (ref) |
| Yes | 20.49 | **3.70 (3.18–4.29)** | **1.97 (1.67–2.31)** |
| **History of suicidality** |  |  |  |
| No | 6.95 | 1 (ref) | 1 (ref) |
| Yes | 182.70 | **25.21 (15.13–42.02)** | **4.98 (2.89–8.58)** |
| **Urgency of surgery** |  |  |  |
| Elective | 6.40 | 1 (ref) | 1 (ref) |
| Non-elective | 9.79 | **1.44 (1.23–1.68)** | **1.20 (1.01–1.42)** |
| **Type of surgery** |  |  |  |
| Breast | 4.86 | **0.56 (0.40–0.80)** | 0.79 (0.55–1.12) |
| ENT | 4.45 | **0.52 (0.29–0.90)** | 0.68 (0.38–1.19) |
| General Surgery | 8.39 | 0.96 (0.76–1.21) | 0.93 (0.73–1.18) |
| Gynecology | 9.06 | 1.05 (0.81–1.37) | 0.86 (0.64–1.14) |
| Neurosurgery | 9.77 | 1.05 (0.60–1.85) | 0.98 (0.54–1.75) |
| Orthopedic | 5.24 | **0.60 (0.46–0.78)** | 0.91 (0.70–1.19) |
| Spine | 8.63 | 1 (ref) | 1 (ref) |
| Thoracic | 5.55 | 0.60 (0.35–1.03) | 0.54 (0.29–1.01) |
| Urology | 4.77 | **0.54 (0.33–0.87)** | 0.85 (0.52–1.39) |
| Vascular | 5.03 | 0.56 (0.30–1.01) | 0.85 (0.46–1.57) |

ENT: ear, nose, and throat (otolaryngology).

Bolded estimates are statistically significant at p<0.05.
